# Transcriptional modification and the accumulation of flavonoid in the leaves of *Cissus rotundifolia *Lam. in respond to drought stress

**DOI:** 10.1007/s44154-024-00205-6

**Published:** 2025-03-10

**Authors:** Qingyun Li, Duncan Kiragu Gichuki, Huimin Zhou, Yujun Hou, Robert Wahiti Gituru, Qingfeng Wang, Haiping Xin

**Affiliations:** 1 CAS Key Laboratory of Plant Germplasm Enhancement and Specialty Agriculture, Core Botanical Gardens/Wuhan Botanical Garden, Chinese Academy of Sciences, No. 201, Jiufeng 1st Road, Donghu New Technology Development Zone, Wuhan, 430074 China; 2 Sino-Africa Joint Research Center, Chinese Academy of Sciences, Wuhan, 430074 China; 3 University of Chinese Academy of Sciences, Beijing, 100049 China; 4 Department of Botany, Jomo Kenyatta University of Agriculture and Technology, Nairobi, 62000-00200 Kenya

**Keywords:** Metabolites, Flavonoid biosynthesis, Drought treatment, Transcription factors

## Abstract

*Cissus rotundifolia* Lam. is an evergreen climber that mainly distributed in the tropical savannas of eastern Africa with excellent drought resistance. While the mechanism underlying drought stress response in *C. rotundifolia* remains unknown. In this study, we investigated the transcriptomics and flavonoid metabolomics responses of *C. rotundifolia* leaves under different drought conditions. We identified a total of 2401 differentially expressed genes (DEGs) in drought-treated leaves. The continuously increasing DEGs were significantly enriched in pathways related to phenylpropanoid biosynthesis, flavonoid biosynthesis, and galactose metabolism. The total flavonoids content were also found increased significantly during drought treatment in the leaves of *C. rotundifolia*. A total of 57 distinct flavonoids were identified using UPLC-MS/MS. Among them, nine out of ten differentially accumulated metabolites (DAMs) displayed notable accumulation profiles under drought conditions. These DAMs included two flavanols [(-)-Catechin gallate and (-)-gallocatechin gallate], two flavonols (myricetin and astragalin), four flavones (orientin, cynaroside, isoorientin, and vitexin), and one flavanone (naringenin-7-glucoside), indicating their pivotal roles in drought response. Additionally, a DEG with continuously high-expression, annotated flavone synthase (*FLS*) synthesises, and synthetizing myricetin under drought stress was tightly related to additional genes belonging to ABA-signaling genes, stomata movement genes, transcription factors, and protein kinases, these genes were contained Top 10 hub-genes of network constructed all DEGs. The results reflect the significance of *FLS* and potentially regulatory genes under drought conditions. These findings suggest that flavonoids play a pivotal role in the drought stress response of *C. rotundifolia*, advancing our understanding of flavonoids accumulation and the transcriptional regulation involved in this process.

**Supplementary Information:**

The online version contains supplementary material available at 10.1007/s44154-024-00205-6.

## Introduction

The rise in the human population has led to an increasing demand for food and medicine. However, the cultivation of crops, vegetables, and medicinal plants is increasingly limited by frequent drought events accelerated by climate change (Fahad et al. 2017). To adapt to drought, plants employ physiological, biochemical, and molecular mechanisms that promote either drought avoidance or tolerance (Gupta et al. 2020; Ahluwalia et al. 2021). Following drought, plants can effectively uptake water via lager and deep root system, enhance water conduction by forming lager cross-section of the vessels and shorter internodes, limit transpiration through progressively closed stoma with abscisic acid (ABA) and declined net photosynthesis in parallel (Farooq et al. 2009). To scavenge reactive oxygen induced by drought-stress, antioxidant system and osmotic regulation play crucial roles in defense responses against this condition. The increased activities of antioxidant enzymes were found in various plants at drought stress (Li et al. 2013; Zarbakhsh et al. 2024), thereby reducing the oxidative damage. When explored to drought, plants could accumulate protective substances to stable membrane system, such as carbohydrates, dehydrins, drought-responsive genes, and even metabolites (Zhang, et al. 2024).

Flavonoids, as the major class of plant secondary metabolites, play pivotal roles in signaling and defense against biotic and abiotic stress agents (Winkel-Shirley, 2001). Flavonoids are synthesized via the conserved phenylpropanoid pathway which involves a range of enzymes (Hodaei et al. 2018). Key enzymes in flavonoids metabolism identified in various plants include chalcone synthase (CHS), chalcone isomerase (CHI), flavanone 3-hydroxylase (F3H), flavonoid 3’-hydroxylase (F3’H), anthocyanin reductase (ANR), and dihydroflavonol 4-reductase (DFR) (Winkel-Shirley 1999). Modification reactions, such as prenylation and methylation, have led to the identification of about 9000 flavonoid compounds in plants, each with diverse functions. Flavonoids are known to mitigate oxidative stress induced by drought (Nakabayashi et al. 2014). The epigallocatechin gallate, epicatechin, and epicatechin gallate in *Cistus clusii* rised progressively in response to drought (Iker et al. 2004). An increased accumulation of flavonoid and phenolic compounds was observed with water deficit *Achillea pachycephalla* Rech. F (Gharibi et al. 2013). Similary, enhanced levels of total phenols, flavonoids, and anthocyanins were noted under drought conditions in wheat (Ma et al. 2014). Various subclasses or metabolites belonging to flavonoids were in response to drought condition.

Expression patterns for key genes associated with flavonoid biosynthesis increase with drought stress were consistent with elevated flavonoid contents (Gharibi et al. 2019). In rice, flavonoid-related genes showed increased expression in response to water deficit, ABA, and salinity (Ithal & Reddy 2004). In wheat, a rapid increase in expression levels of flavonoid-related genes, including *CHS*, *CHI*, *FLS*, *DFR*, *ANS*, and *FNS*, were reported under drought condition (Ma et al. 2014). A comparative study involving two cultivars of *Chrysanthemum morifoilum* subjected to drought revealed cultivar-specific patterns of flavonoids accumulation and expression of related key genes (Hodaei et al. 2018). A set of transcription factors, including the MYB, bHLH, and WD40 families, has been shown to control flavonoids production (Schaart et al. 2013; Xu et al. 2015). MYB transcription factor encoding genes were found observed differential expression and accumulation of flavonoids-related genes in *Anoectochilus roxburghii* (Chen et al. 2020a, b). The MYB and FLS genes have similar expression patterns, indicating a crucial role of the observed tissue-specific accumulation of rutin (Liang et al. 2019). MYB-bHLH-WDR complexes conserved in higher plants were involved in different types of transcriptional regulation of flavonoid biosynthesis pathway by positive feedback (Xu et al. 2015).

*Cissus rotundifolia*, an evergreen climber in the grape family, thrives in tropical savannahs that experiences periodic water scarcity, resulting in its higher drought tolerance. This species has been widely utilized in traditional medicinal preparations and has also been introduced to other regions as an ornamental plant. The succulent leaves and facultative crassulacean acid metabolism photosynthesis are favour with drought adaptive evolution of *C. rotundifolia* (Xin et al. 2022). Recently, Gichuki et al. (2021) reported approximately 220 flavonoid-related metabolites in three tissues of *C. rotundifolia* and examined the flavonoid concentration across these tissues. But the drought response and its regulation mechanism in this species remain unexplored.

Our study aimed to explore the drought responses and regulatory mechanism of *C. rotundifolia* through integrating transcriptome and metabolome. Firstly, drought responses of its leaves were observed at transcriptional levels. A serious of differentially expressed genes (DEGs) with continuous increase was enriched in flavonoid biosynthetic pathway genes in *C. rotundifolia* under varying water deficiency condition. Additionally, increased total flavonoids content and 57 various flavonoids content were detected under drought stress by aluminum chloride colorimetric method and the flavonoid metabolome respectively. Among them, 10 kinds of differential accumulated metabolites (DAMs) were noticed to positively regulate drought response in *C. rotundifolia* leaves. Further, we infer potential regulatory genes associated with flavonoids biosynthesis, transcription factors, ABA biosynthesis and signaling pathway, photosynthesis, stomatal movement, and antioxidant enzyme. The findings will provide a foundation for understanding the drought tolerance response in *C. rotundifolia*, which could potentially be engineered to enhance drought tolerance in other members of grape family.

## Results

### Phenotypic and physiological responses of *C. rotundifolia* leaves under drought stress

**Fig. 1:**
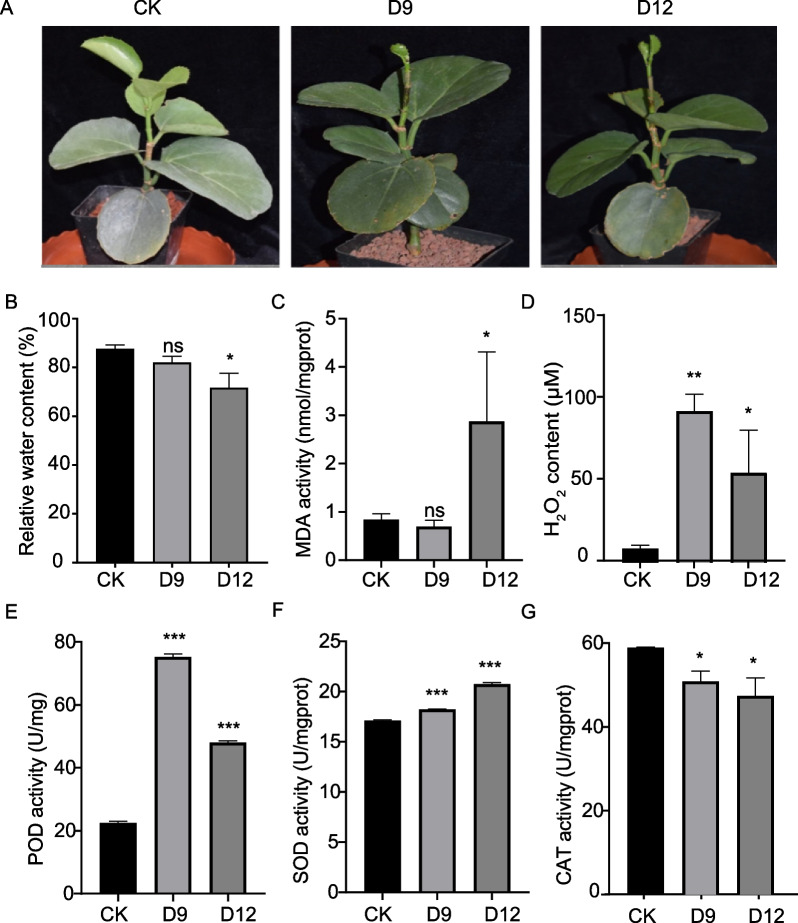
The phenotype and physiological changes in the leaves of *C. rotundifolia* under drought stress treatment. **A** the cutting of *C. rotundifolia* subjected to control and drought treatments. **B** Relative water content (RWC) in the leaves under control and drought treatments. The contents of (**C**) MDA and (**D**) H_2_O_2_ in *C. rotundifolia* leaves under drought treatment. **E**–**F** The enzyme activities of POD, SOD, and CAT, respectively. Data represent mean values ± *SE* of three biological replicates. CK, D9, D12 represent 100%, 10% and 0% soil water content, respectively. CK group was used as the control. Significant differences between each treatment and the control are indicated by *t*-test (* *p* < 0.05, ** *p* < 0.01, *** *p* < 0.001, ns *p* > 0.05)

To simulate the arid environments, *C. rotundifolia* cuttings were exposed to medium with 100% (CK: control), 10% (D9: 9 days after watering), and 0% (D12: 12 days after watering) relative water contents, respectively. The plants didn’t show obvious phenotypes such as wilting or yellowing of the leaves even under severe drought at D12 (Fig. 1A). The relative water content (RWC) in leaves didn’t change at D9 when RWC in medium dropped to only 10%. And the RWC in leaves declined to 70% at D12 with almost no water in the medium (Fig. 1B). These results indicate the excellent drought stress tolerance of *C. rotundifolia*.

The MDA content was measured to evaluate the level of membrane lipid peroxidation during drought treatment. As shown in Fig. 1C, the MDA only increased at D12, consistent with the trend of RWC in leaves, which indicate that the damage of membrane occurs only in severe drought conditions in the leaves of *C. rotundifolia*. While the content of H_2_O_2_ increased both in D9 and D12 comparsions with control (Fig. 1D). Additionally, the activities of antioxidant enzymes (POD and SOD) increased after drought treatment (Fig. 1E and F), whereas the activities of CAT decreased significantly (Fig. 1G). These results indicated that the plants indeed sensed the drought stress at D9 before it loss water and show the damage at D12, although no obvious morphological change can be observed. The stress condition at D9 was defined as moderate drought stress and the D12 as severe drought stress based on the RWC in leaves and stress related physiological indexes.

### Transcriptome analysis for *C. rotundifolia* under drought stress

To investigate the transcriptional response toward to drought stress in *C. rotundifolia*, the RNA-seqs were conducted on 9 leaves samples including CK, D9, and D12 with 3 biological replications, respectively. A total of 16,162 genes (52.4% of the total annotated genes in *C. rotundifolia* genome) were identified with their expression detected at least in one of the samples. Among them, 1246, 1849, 1474 DEGs were identified in CK vs. D9, CK vs. D12, and D9 vs. D12, respectively (Table S1).

**Fig. 2:**
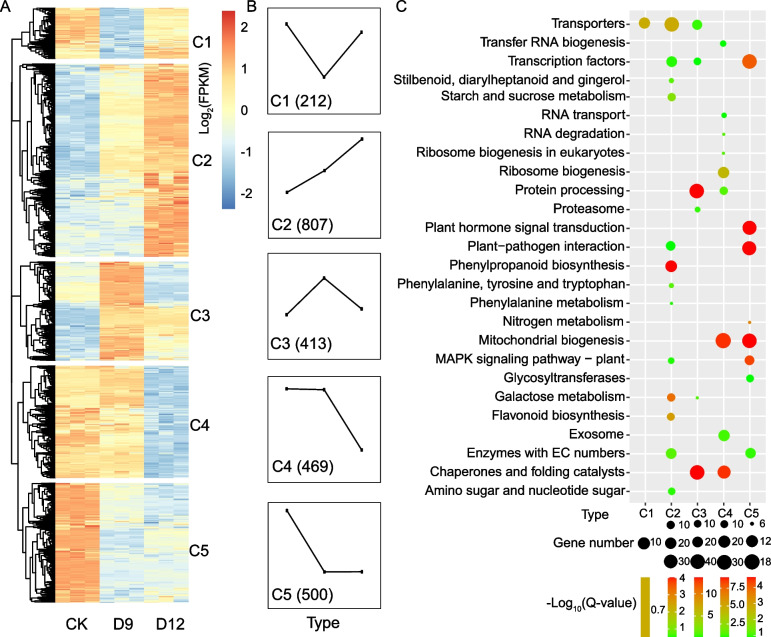
Differentially expressed genes (DEGs) in *C. rotundifolia* leaves under progressive drought treatment. **A** DEGs clustered into five groups based on their abundance levels. Genes with |Log_2_FC|> 1, *q*-value < 0.05, and at least one value > 1 among two comparison groups (D9 vs CK, D12 vs CK, D12 vs D9) were considered as DEGs. Clusters are denoted as “C1-C5”. **B** Summary of the expression patterns for the five clusters. **D** KEGG enrichment analysis of DEGs in the five clusters

Then all the DEGs were divided into five distinct clusters according to its expression patterns by hierarchical clustering analysis (Fig. 2A and B). In cluster 1, decreased expression levels were observed only under drought conditions at D9. These DEGs were mainly located in the apoplast and involved in defense response (Table S2). Cluster 2 contained the most DEGs (807) that were up-regulated during drought treatment, suggesting that these drought-responsive genes play important role for drought stress. Gene ontology (GO) results showed these genes were mainly enriched in terms such as “response to water deprivation”, “response to acid chemical”, and “plasma membrane” (Table S2). KEGG enrichment shows that the DEGs in cluster 2 were primarily involved in phenylpropanoid biosynthesis, flavonoid biosynthesis, and galactose metabolism pathways (Fig. 2D). Cluster 3 contained early drought-responsive genes that only up-regulated at D9, which were enriched in “response to oxidative stress”, “response to temperature stimulus”, and “response to oxygen-containing compound”. Genes in cluster 3 were overrepresented in “protein processing in endoplasmic reticulum”, “chaperones and folding catalysts”, and “galactose metabolism” (Fig. 2D). DEGs in clusters 4 were composed by down-regulated genes in severe drought conditions. Enriched pathways on mitochondrionand ATP binding) suggest that these genes involving in energy metabolism might protect organization through reducing consumption to overcome severe condition. Cluster 5 represented genes that were inhibited under drought stress (Fig. 2C). Thus, plant hormone and signaling transduction pathways were the first to be affected for drought stress (Table S2). These results indicate that the varied expression profiles of genes could be part of the drought response mechanism in this species.

### Drought-responsive pathways in *C. rotundifolia*

**Fig. 3:**
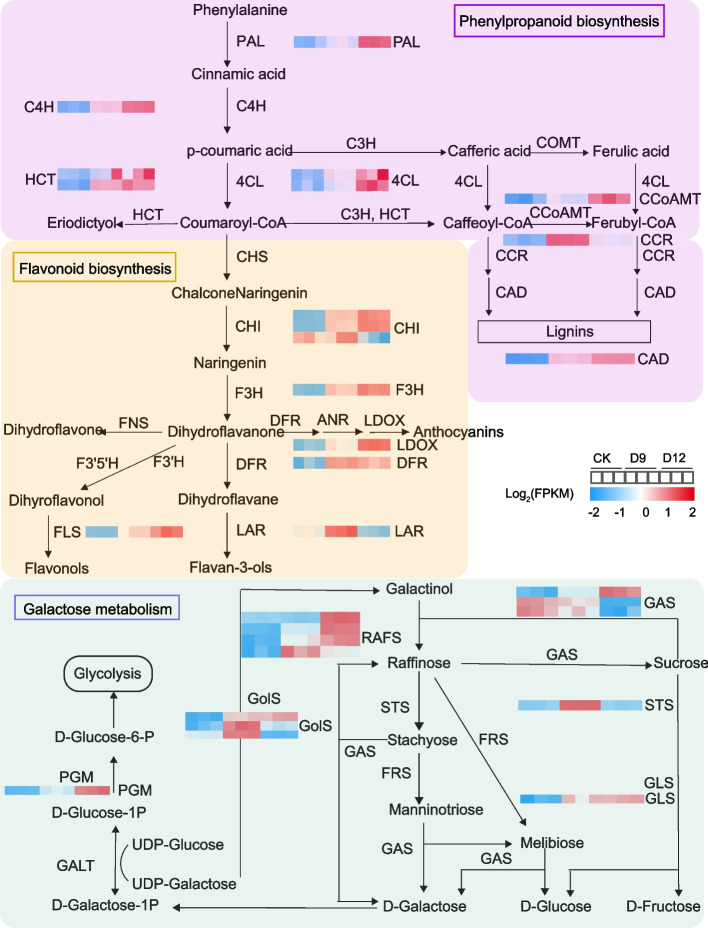
Expression levels of genes in phenylpropanoid biosynthesis, flavonoid biosynthesis, and galactose metabolism pathways under different drought treatments (CK, D9, D12) in *C. rotundifolia* leaves. Enzymes abbreviations: PAL, phenylalanine/tyrosine ammonia-lyase; C4H, trans-cinnamate 4-monooxygenase; 4CL, 4-coumarate–CoA ligase; C3H, p-coumarate 3-hydroxylase; HCT, shikimate O-hydroxycinnamoyltransferase; COMT, caffeic acid 3-O-methyltransferase/acetylserotonin; CCoAMT, caffeoyl-CoA O-methyltransferase; CCR, cinnamoyl-CoA reductase; CAD, cinnamyl-alcohol dehydrogenase; CHS, chalcone synthase; CHI, chalcone isomerase; F3H, flavonoid-3- hydroxylase; DFR, bifunctional dihydroflavonol 4-reductase/flavanone 4-reductase; LAR, leucoanthocyanidin reductase; F3'H, flavanone-3'-hydroxylase; F3',5'H, flavonoid 3',5'-hydroxylase; FNS, flavone synthase; FLS, flavonol synthase; ANR, anthocyanidin reductase; LDOX, leucoanthocyanidin dioxygenase. RAFS, raffinose synthase; GAS, alpha-galactosidase; STS, stachyose synthetase; FRS, beta-fructofuranosidase; GLS, alpha-glucosidase; GolS, galactinol synthase; GALT, UDPglucose–hexose-1-phosphate uridylyltransferase; PGM, phosphoglucomutase (alpha-D-glucose-1,6-bisphosphate-dependent)

To better understand the key drought-responsive pathways in Cluster 2, genes involved in the accumulated biological processes including phenylpropanoid and flavonoid biosynthesis, as well as galactose metabolism were identified and visualized in Fig. 3. A total of 104 key genes encoding 25 enzymes involved in these pathways were identified in *C. rotundifolia* (Table S3). After excluding genes with lower expression (41 genes, FPKM < 1), nearly half of remaining genes (30/63) exhibited differential expression under early or/and late drought treatment (Fig. 3). A general increase in the expression levels of key genes in these three pathways was observed with increasing water deficit. In phenylpropanoid biosynthesis, DEGs belonging to the phenylalanine/tyrosine ammonia-lyase (PAL), trans-cinnamate 4-monooxygenase (C4H), 4-coumarate–CoA ligase (4CL), shikimate O-hydroxycinnamoyltransferase (HCT), cinnamoyl-CoA reductase (CCR), caffeoyl-CoA O-methyltransferase (CCoAMT), and cinnamyl-alcohol dehydrogenase (CAD) gene families had higher expression in drought treatments compared to CK. In flavonoid biosynthesis, eight DEGs [three CHIs, one F3H, one leucoanthocyanidin dioxygenase (LDOX), one DFR, one flavonol synthase (FLS), and one leucoanthocyanidin reductase (LAR)] were identified, with most (7/8) being highly expressed under drought stress. Additionally, eleven out of thirteen DEGs involved in galactose metabolism in *C. rotundifolia* were significantly up-regulated under in response to early or/and drought response, particularly genes in the raffinose synthase (RAFS) gene family, which showed higher expression. Overall, almost all DEGs (26/30) in the phenylpropanoid biosynthesis, flavonoid biosynthesis, and galactose metabolism pathways were up-regulated in response to drought stress, demonstrating their contributions on drought stress in *C. rotundifolia.*

### Metabolomics analysis for *C. rotundifolia* under drought stress

**Fig. 4:**
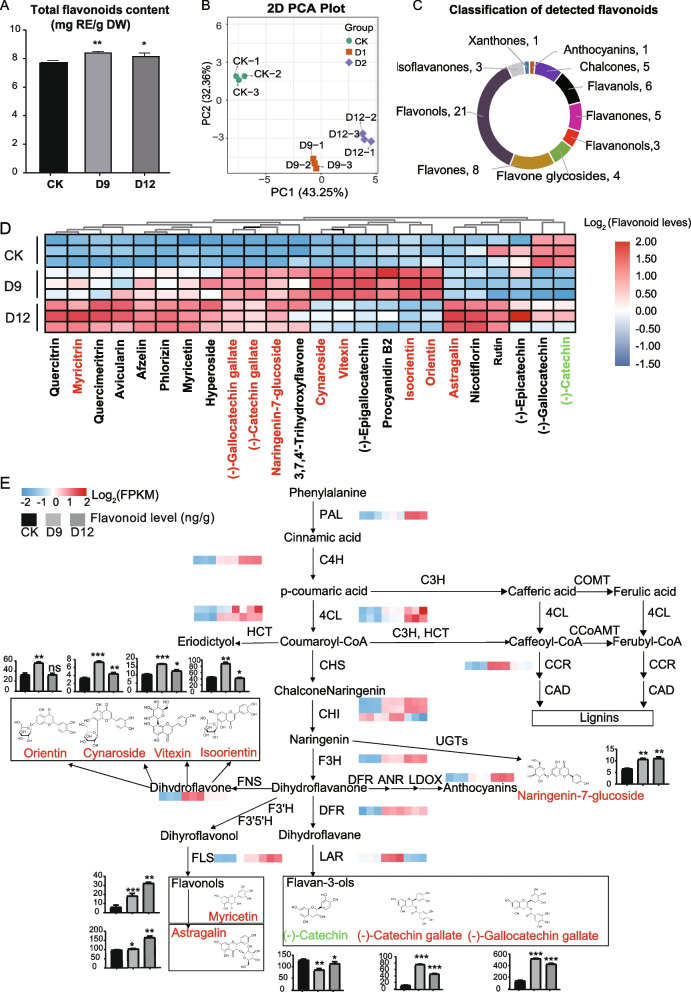
Metabolomics analysis of *C. rotundifolia* under drought stress. **A** Total flavonoids content under drought treatment. Data represent mean values ± SE of three biological replicates. CK, D9, D12 correspond to 100%, 10% and 0% soil water content, respectively. The CK group was used as the control. Significant differences between each treatment and the control are indicated by t-test (* *p* < 0.05, ** < *p* < 0.01). **B** Principal component analysis (PCA) score plot of mass spectrometry data for each group. The x-axis and y-axis represent the first and second principal components, respectively. The percentage represents the contribution of the principal component to the sample variance. **C** Classification of detected flavonoid metabolites. The bars represent the flavonoid subclasses, and the numbers indicate the respective compounds within each subclass. **D** Cluster analysis of detected flavonoid metabolites that flavonoid levels > 3 ng/g across the three groups. Up-regulated or down-regulated differentially accumulated metabolites (DAMs) were highlighted by red or green color. **E** Integration of DEGs and DAMs in flavonoid biosynthesis pathway. Blue color of heat-map indicates low gene expression and the red indicates high gene expression. Column charts represented flavonoid contents at CK, D9, and D12

Transcriptome analysis revealed that flavonoid biosynthesis was enriched during drought stress in *C. rotundifolia.* Thus, we firstly examined the total flavonoid content of leaves under drought stress using colorimetric method. Higher level of total flavonoid content was detected under moderate drought (D9) and severe drought (D12) compared to plants under normal conditions (CK). The flavonoid content ranged between 7.79, 8.45, and 8.22 (mg RE/g DW) for CK, D9, and D12 respectively (Fig. 4A).

To further explore the types of flavonoids accumulated on drought stress, multiple flavonoid profiles in *C. rotundifolia* leaves were examined using high-performance liquid chromatography (HPLC). Principal component 1 of the compounds (43.25%) exhibited clear distinct patterns between control and drought groups (Fig. 4B). The samples were well-grouped, along with their biological replicates. According to the total ion flow chromatography overlap and quality control (QC) samples, the instruments showed high reliability and reproducibility. The retention time and peak intensity for the QC sample remained constant in the three test samples (Figure S1). Qualitative and quantitative analysis of the mass spectrometry data from drought stress samples was conducted based on the self-built MetWare database (MWDB). A total of 57 compounds including anthocyanins, chalcones, flavanols, flavanones, flavononols, flavone glycosides, flavones, flavonols, isoflavanones, and xanthones were detected and quantified (Table S4, Fig. 4C). Flavonols were the most abundant, followed by flavones, and have been associated with higher antioxidant properties in other plant species.

Variations in flavonoid accumulation levels were identified from 24 metabolites grater than 3 ng/g under drought conditions (Fig. 4D). The accumulation levels of most flavonoids increased with the severity of the water deficit. The majority of metabolites (16/24) exhibited higher accumulation level at the D12 sampling point, representing one-week of treatment at zero soil water content. Among them, the accumulation levels for flavonol compounds (10/16) were generally high under severe drought conditions (D12).

Ten DAMs more intuitively demonstrated the content differences between groups (abundance > 3, |Log2(Fold change)|> 0.58, VIP > 1, and *Q*-value < 0.05) (Fig. 4D and E). Most DAMs (9/10) including two flavanols [(-)-Catechin gallate and (-)-gallocatechin gallate], two flavonols (myricetin and astragalin), four flavones (orientin, cynaroside, isoorientin, and vitexin), and one flavanone (naringenin-7-glucoside), exhibited increased in flavonoid levels with increasing water deficit compared to CK. Only (-)-catechin showed decreased abundance in D9 and D12, and isoorientin showed decreased in D12 compared to CK.

### Candidate flavonoid biosynthesis-related DEGs under drought stress

All DEGs under drought stress were used to construct gene co-expression network, where the top 10 hub genes included one aldehyde oxidase (AAO), two 9-cis-epoxycarotenoid dioxygenases (NCEDs), two stomata-related genes, three transcript factors (TFs: MYB, bZIP, and WD40), and one receptor kinase genes (Figure S2 and Table S3, Table S5).These findings suggest that these ten genes played important roles drought stress response in the leaves of *C. rotundifolia*.

Combining the two sets of omics data, multiple candidate flavonoid biosynthesis-related genes in *C. rotundifolia* were explored under drought stress. Key genes in the flavonoid biosynthesis pathway, especially those acting at the end of reactions and showing differential expression such as *FLS* (*CRGY0215161*) and *LAR* (*CRGY0211581*), might be core regulators for flavonoid biosynthesis in response to drought stress (Fig. 4E and Figure S3).

**Fig. 5:**
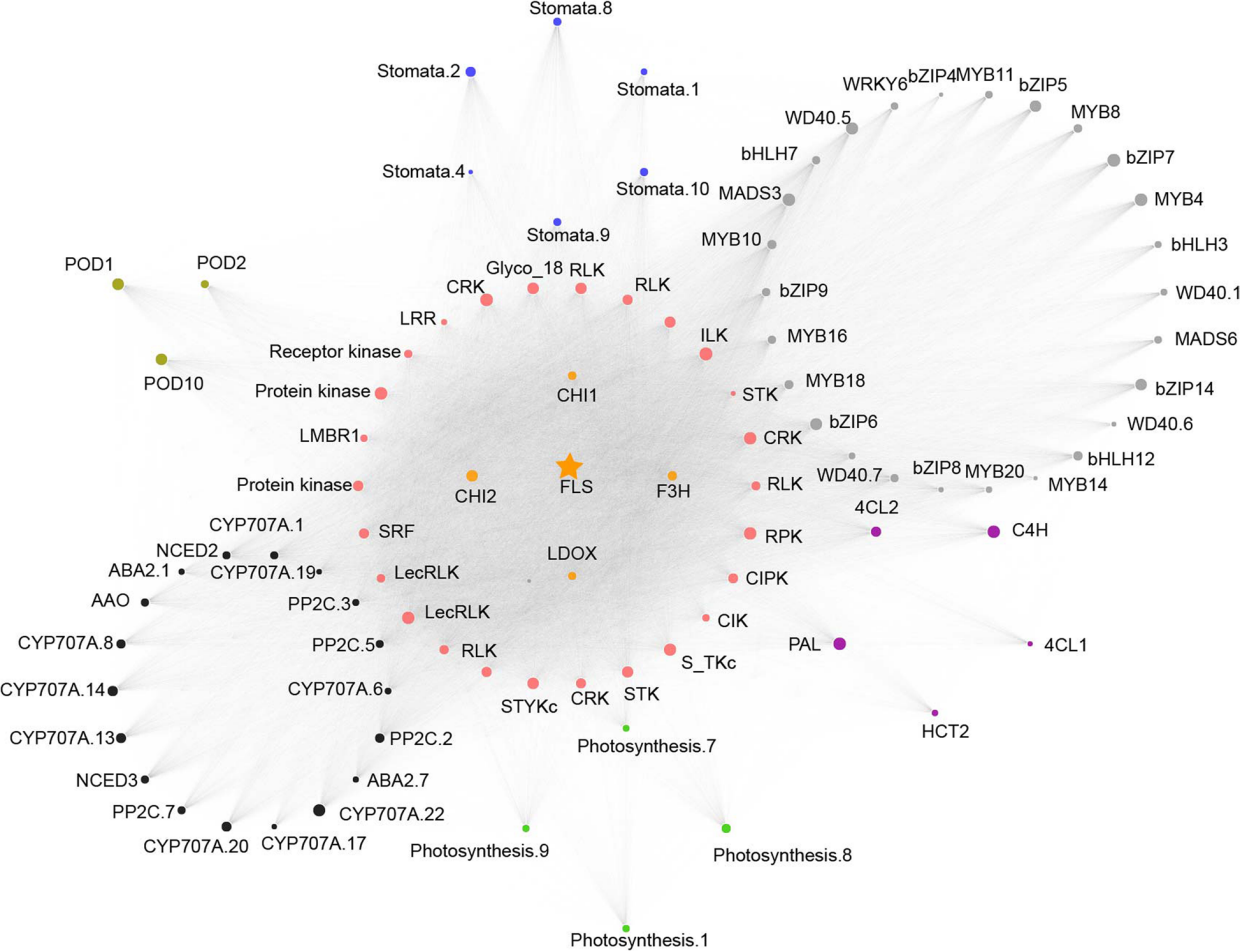
DEGs directly interacted with *FLS* (*CRGY0215161*). The orange, grey, black, green, blue, brown, and pink circles represent DEGs in flavonoids biosynthesis, transcription factors, ABA biosynthesis and signaling pathway, photosynthesis, stomatal movement, antioxidant enzymes and protein kinases, respectively. The size of circles represents the connectivity of the genes

To investigate more flavonoid biosynthesis-related DEGs, we identified 77 TFs including 23 MYBs, 14 bHLHs, 8 WD40s, 14 bZIPs, 12 WRKYs, and 6 MADXs are DEGs in *C. rotundifolia* under drought conditions (Table S3). As key enzymes in the final steps of flavonoid biosynthesis, *FLS* and *LAR* are typically involved in forming flavonols (myricetin and astragalin), and flavanols [(-)-catechin, catechin gallate, and (-)-gallocatechin gallate] under drought treatments in *C. rotundifolia* (Fig. 4E). Co-expression network showed these two genes are positively related to *MYBs*, *bZIPs*, *bHLHs*, *WD40s* (Fig. 5 and Figure S4). *FLS* (*CRGY0215161*) was positively connected with more DEGs, including 4 flavonoid-related genes, 18 ABA-related genes, 26 TFs, 6 stomata-related genes, 3 POD, and 26 protein kinases (Fig. 5 and Table S6). However, *LAR* (*CRGY0211581*) interacted with 1 CCR, 7 TFs (1 MYB, 3 bHLHs, 1 bZIP, 1 WRKY, and 1 WD40), 4 ABA-related genes, 1 POD, and 1 SOD gene (Figure S4). Interestingly, top 10 hub genes from network constructed by of all DEGs were observed in net of FLS, further indicating the significance of these genes and flavonoid synthesis pathway under drought. But no interacted-gene was shared between two networks (connected with *FLS* and *LAR*). These results indicate that specific TFs (bZIPs, MYBs, WD40) might regulate the differential expression of structural genes (flavonoid-, ABA- related, POD genes) under drought stress, providing insights into potential candidate regulated factors implicated in flavonoid metabolites biosynthesis in *C. rotundifolia* and their possible role in the drought stress response.

## Discussion

*Cissus rotundifolia* exhibits exceptional survival under drought conditions and is widely used for medicinal purposes as well as a vegetable in some communities. Biological and environmental factors contribute to the accumulation of secondary metabolites, which are associated with various biological functions (Ali 2014). Flavonoids are a diverse class of secondary metabolites with a wide range of effects on plant physiology. Up to now, flavonoids could enhance drought resistance of plants via increasing antioxidant activities (Mishra et al. 2023). For example, overaccumulation of antioxidant flavonoids was found to enhance oxidative and drought tolerance in Arabidopsis (Nakabayashi et al. 2014). Flavonoids may improve drought tolerance of maize seedling by reducing oxidative damage and regulating stomatal movement (Li et al. 2021). Increased total flavonoid content in pea under salt or drought stress shows important roles in antioxidant activitie (Farooq et al. 2021). The accumulation of flavone under drought condition was also found in *Sorghum bicolor* along with adaptive shift from lignin biosynthesis to flavonoid pathway (Fontanet-Manzaneque et al. 2024). As observed in our study, drought stress lead to increased flavonoid levels in *C. rotundifolia* leaves, which likely enhances their antioxidant properties.

*FLS*, *F3H*, and *LAR* are competing for enzymes that use a similar flavanone substrate. Among the key enzymes identified in the flavonoid biosynthesis pathway, *CHS* in notably related to drought stress. In *Solanum tuberosum* varieties, the *CHS* gene exhibited maximum expression under severe drought stress (Vasquez-Robinet et al. 2008). Similarly, in wheat, an up-regulation trend has been reported for both *CHI* and *CHS* genes under drought stress, suggesting their protective roles in response to drought (Ma et al. 2014). The *LAR* gene (*pycom13g04100*) exhibited the highest expression level at the lowest-temperature cold treatment in *Pyrus hopeiensis* flowers (Li et al. 2023). *LAR* (*TEA027582*) in *Camellia sinensis* was also up-regulated under drought treatment. Soil drought could induce the gene expression of LAR, leading the accumulation of catechins (Song et al. 2016). On the other hand, *F3H* transcripts expression levels were upregulated in grapefruits under limited water stress (Castellarin et al. 2007). Although limited studies have indicated the role of *FNS* in abiotic stress, our study showed that *FNS* transcripts level increased with drought severity, similar findings by Ma et al. (2014). Generally, transcripts for the late branched section of the flavonoid biosynthesis pathway are enhanced by drought stress in grapes and *Populous euramericana* fruits (Castellarin et al. 2007; Kim et al. 2012). In our study, relative expression profiles for *FLS*, *DFR*, and *ANS* were upregulated with increased drought severity.

The impacts of biotic stresses on the flavonoid biosynthesis pathway have been explored in many plants, including crops such as rice (Ithal & Reddy 2004) and wheat (Liu et al. 2013a), implicating them in drought stress response mechanisms. Flavonoids act as antioxidants by halting the production of reactive oxygen species (Agati & Tattini 2010) and neutralizing of formed ROS (Jaakola & Hohtola 2010). In this study, although all treatments had approximately similar compounds, variations in their concentration were noted with increasing drought severity. The increase was observed for most flavonols, which are associated with higher antioxidant efficiency. Additionally, anthocyanins are known for their strong antioxidant properties due to their higher hydroxylation abilities. However, only one anthocyanin was detected in our study, possibly due to limitations in the current assays that might not detect compounds present in trace amounts. Considering the vast number of flavonoids detected in plants, we speculate that more flavonoids could be involved in drought response mechanism in this species. Therefore, further studies should be conducted to explore the biochemical and regulatory mechanisms utilized by this species in response to drought stress.

Flavonoids and their sub-classes are synthesized through the conserved phenylpropanoid pathway, which is regulated by numerous structural genes (Liu et al. 2013b). The role of the main structural genes involved in flavonoids biosynthesis in *C. rotundifolia* has previously been described by Gichuki et al., (2021). Flavonoids biosynthesis is regulated by MYB family transcription factors, among others, including bHLH and WD40 proteins (Lloyd et al. 2017; Naik et al. 2022). The change of flavonoid may facilitate drought tolerance through the regulation of MYB-WD40-bHLH complex in grafted potatoes (Jian et al., 2024). The overexpression of MdMYB88 and MdMYB124 has been shown to enhance drought resistance in apple by regulating the abundance of phenylpropanoids and flavonoids (Geng et al. 2020). In S*alvia miltiorrhiza* bunge, *SmWD40-170,* a gene responsible for drought tolerance, medicates drought resistance by regulating the stomatal movement (Liu et al. 2020). FlbZIP12 in *Fagopyrum leptopodum* promotes drought resistance by interacting with FlSnRK2, resulting in higher expression levels in the flavonoid biosynthesis pathway (Wang et al., 2023). Although our results suggest a coordinated relationship between the transcription factors and the flavonoid accumulation, further validation is needed to confirm this hypothesis.

## Conclusion

In summary, flavonoid metabolomics and transcriptomics were conducted in leaves of *C. rotundifolia* under drought stress. The flavonoid contents increased with the duration of drought treatment under severe drought conditions, consistent with the higher expression profiles of key structural genes in the pathways of phenylpropanoid and flavonoid biosynthesis, such as metabolites (myricetin and astragalin) and their synthesized genes (*FLS* and *LAR*). Meanwhile, the potential regulatory genes such as *FLS*, *LAR* and other TFs were also identified. Our findings enhance the understanding of flavonoid biosynthesis in *C. rotundifolia* and provide a foundation for further studies on drought stress response mechanisms in this species and other members of the grape family.

## Materials and methods

### Plant materials and drought treatments

Stem cuttings of *C. rotundifolia* were collected from Endau hill, Kitui County, Kenya. The plants were then cultivated in the greenhouse at Wuhan Botanical Garden, Chinese Academy of Sciences. The cuttings were grown from stem-cutting in a mixture of nutrient soil and vermiculite (soil ratio of 2:1). Cuttings that developed roots and had 3–4 mature leaves were selected for drought treatments. Selected cuttings were transferred to new pots with volcanic stone as medium to accelerate the water loss during drought treatment. The total weight of the pots, the medium, and cuttings was recorded before watering. Then the plants were fully watered, and the weights were measured to determine the water holding capacity of the medium. The transferred cuttings were well watered for one week to acclimate in the new medium. For drought treatment was performed by stopping watering. The water content in the mediums was calculated twice daily, and samples were collected at 9 and 12 days after drought treatment when mediums holds approximately 10% and 0% of waters when compared with its maximum water hold capacities, respectively. The well watered samples were used as controls. Mature leaf samples were collected early in the morning before sunrise, frozen in liquid nitrogen, and stored at −80 °C for RNA extraction and metabolome analysis. Additionally, fresh samples were taken for relative water content determination and physiological assays. Sampling was carried out in triplicates for each time point.

### Physiological analysis for leaves of *C. rotundifolia* under drought treatment

Fresh weight (FW) of leaves was measured (González and González-Vilar, 2001). Then the leaves were re-weighted (Turgid weight, Tw) after rehydrated in distilled waters for 24 h in darkness at 4 °C, and subsequently oven-dried at 50 °C to a constant weight (Dry weight, Dw). Relative water content was calculated as: Relative water content (RWC, %) = [(Fw-Dw)/(Tw-Dw)] × 100.

Total flavonoid content (TFC) was measured using aluminum chloride colorimetric methods as described by Gichuki et al. (2021). Oven-dried leaves from control and drought treatments were used. Rutin was used to calibrate the standard curve, and the flavonoid contents were expressed as milligrams of rutin equivalents per gram of leaf dry weight.

To prepare crude enzyme extract, 0.2 g of fresh *C. rotundifolia* leaves were homogenized in a pre-cooled mortar with 4 mL of 150 mM phosphoric acid buffer solution (PBS, pH 7.0, containing 1% PVP, added just before use) that pre-cooled at 4℃. Quartz sand was added to aid grinding. The homogenate was centrifuged at 18,514 g for 10 min at 4℃, then the supernatant was collected and used for the measuring of MDA content, H_2_O_2_ content, and enzyme activities for POD and SOD by assay kits (A003-1, A064-1, A084-3, and A001-3 for MDA, Nanjing Jiancheng Bioengineering Institute, Nanjing, China) according to the instructions of the manufacturer.

### RNA extraction, cDNA library construction and RNA-seq

Total RNAs was extracted from *C. rotundifolia* leaves under control and drought treatment using a general plant total RNA extraction kit (BioTeke Corporation., Ltd. cat. NO RP3301, Wuxi, China). The RNAs were quantified using a NanoDrop™ OneC spectrophotometer (Thermo Fisher Scientific Inc., Waltham, USA), and the quality was confirmed by agarose gel electrophoresis. Oligo(dT)-attached magnetic beads were used to purify mRNA, which was then fragmented into small pieces with a fragmentation buffer. First-strand cDNA was synthesized using random hexamer-primed reverse transcription, followed by second-strand cDNA synthesis. A-Tailing Mix and RNA Index Adapters were added for end repair, and the cDNA fragments were amplified by PCR. The products were purified with Ampure XP Beads and dissolved in EB solution. Quality control was performed using the Agilent Technologies 2100 bioanalyzer. The double-stranded PCR products were heat-denatured and circularized to form the final library. Single-strand circle DNA (ssCir DNA) was amplified to create DNA nanoballs (DNBs), which were loaded into the patterned nanoarray and sequenced on the BGIseq500 platform (BGI-Shenzhen, China) to generate paired-end 150 base reads (Goodwin et al. 2016).

Raw data quality was assessed using FastQC software (https://www.bioinformatics.babraham.ac.uk/projects/fastqc/). Clean data were obtained by trimming low-quality reads and adaptors using Trimmomatic v0.36 software. Clean reads were mapped to the reference genome of *C. rotundifolia* (Xin et al. 2022) using TopHat v2.2.1 software (Trapnell et al. 2012). Fragments per kilobase of exon model per million mapped fragments (FPKM) of each gene were calculated using Cufflinks v2.2.1 software (Trapnell et al. 2012)*.* Comparisons among CK vs D9, CK vs D12, and D9 vs D12 were performed using Cuffdiff v2.2.1 (Trapnell et al. 2012). DEGs were defined as having at least one FPKM > 1, |Log_2_(Fold change)|> 1, and false discovery rate (FDR) < 0.05.

### Co-expression network construction

All DEGs were used to construct the co-expression network based on the gene expression under drought stress using Cytoscape v3.7.1 software (Shannon et al. 2003). DEGs involved in flavonoids biosynthesis, transcription factors, ABA biosynthesis and signaling pathway, photosynthesis, stomatal movement, and antioxidant enzyme were obtained by blasting against with orthologous protein sequences in Arabidopsis with an e-value less than 1e^−5^ (Camacho et al. 2009). The correlation coefficient (r) and *P*-value (*p*) between genes were calculated using the “cor” and “cor.test” functions in R. Co-expression gene pairs were selected based on the criteria: r > 0.85 and *p* < 0.0001.

### Flavonoids quantification and profiling using UPLC-MS/MS

Flavonoid contents were detected by MetWare (http://www.metware.cn/) based on the AB Sciex QTRAP 6500 LC–MS/MS platform. Firstly, metabolites of leaves were extracted from freeze-dried, ground using a mixer mill (MM 400, Retsch) with a zirconia bead for 1.5 min at 30 Hz. 20 mg of the sample powder was weighed and extracted with 0.5 mL of 70% methanol (v/v). An internal standard (10 μL, 4000 nmol/L) was added for quantitation. The extract was sonicated for 30 min and centrifuged at 12,000 g for 5 min at 4 °C. The supernatant was collected and the extraction repeated. Sample extracts were analyzed using a UPLC-ESI–MS/MS system (UPLC, ExionLC™ AD: https://sciex.com.cn/; MS, Applied Biosystems 6500 Triple Quadrupole, https://sciex.com.cn/). The analytical conditions were: UPLC column, Waters ACQUITY UPLC HSS T3 C18 (100 mm × 2.1 mm i.d, 1.8 µm); solvent system, water with 0.05% formic acid (A) and acetonitrile with 0.05% formic acid (B); gradient elution: 0–1 min, 10%—20% B; 1–9 min, 20%—70% B; 9—12.5 min, 70%—95% B; 12.5—13.5 min, 95% B; 13.5–13.6 min, 95%—10% B,13.6—15 min, 10% B; flow rate: 0.35 mL/minute; temperature: 40 °C; injection volume: 2 μL.

Mass spectrometry analysis was performed as described by Chen et al., (2013). Linear ion trap (LIT) and triple quadrupole (QQQ) scans were acquired on a triple quadrupole-linear ion trap mass spectrometer (QTRAP), QTRAP® 6500 + LC–MS/MS System equipped with an ESI Turbo Ion-Spray interface, operating in positive and negative ion mode, and controlled by Analyst 1.6.3 software (AB Sciex, Framingham, MA, USA). Flavonoids were analyzed using scheduled multiple reaction monitoring (MRM). Data acquisitions and quantitative analysis were performed using Analyst 1.6.3 (AB Sciex, Framingham, MA, USA) and Multiquant 3.0.3 (AB Sciex, Concord, Ontario, Canada), respectively.

Metabolite identification and annotation were based on the self-built MWDB database (MetWare Biological Science and Technology Co., Ltd. Wuhan, China). Repetitive signals for K^+^, Na^+^, NH_4_^+^ and other heavy molecular weight substances were eliminated during the analysis. Quantification was completed by MRM of triple quadrupole mass spectrometry. Primary and secondary MS data were qualitatively analyzed by comparing the accurate precursor ions (Q1), product ions (Q3), and retention times (RT). After obtaining the mass spectrometry data of different samples, the chromatographic peaks of all target substances were integrated and analyzed quantitatively through the standard curve. MultiQuant 3.0.3 software (AB Sciex, Concord, Ontario, Canada) was used to process the mass spectrometry data (Fraga et al. 2010), referencing to the retention time and peak type information of the standard, to integrate and correct the mass spectrometry peaks detected in different samples, ensuring the accuracy of qualitative and quantitative analysis. Quantification was conducted based on the external standard curve calibration from the respective synthetic standards with concentrations ranging from 0.5–2000 nmol/L. From the calculated slope obtained using the calibration curve, the concentration for the flavonoids was determined.

### Metabolome data analysis

PCA for the cleaned data was performed by prcomp function within R v3.5.1 (Lever et al. 2017). Hierarchical clustering for samples and metabolites was carried out and presented as heatmaps using TBtools (Chen et al. 2020a, b). DAMs were identified by the criterias of flavonoid content > 3 ng/g, Log_2_|Fold change|≥ 0.58, and variable importance in project (VIP) ≥ 1 using R package “metaboAnalystR”. VIP values were obtained from OPLS-DA analysis (Thevenot et al. 2015).

## Supplementary Information

Supplementary Material 1.

Supplementary Material 2.

## Data Availability

All data used and generated in this study have been deposited in the National Genomics Data Center (https://ngdc.cncb.ac.cn/) with the project number PRJCA031303.

## Abbreviations

D12: 12 Days after watering/Severe drought
4CL: 4-Coumarate–CoA ligase
D9: 9 Days after watering/Moderate drought
NCED: 9-Cis-epoxycarotenoid dioxygenase
ABA: Abscisic acid
Q1: Accurate precursor ions
AAO: Aldehyde oxidase
ANR: Anthocyanin reductase
CCoAMT: Caffeoyl-CoA O-methyltransferase
CHI: Chalcone isomerase
CHS: Chalcone synthase
CCR: Cinnamoyl-CoA reductase
CAD: Cinnamyl-alcohol dehydrogenase
CK: Control/Normal conditions
c: Correlation coefficient
DAMs: Differentially accumulated metabolites
DEGs: Differentially expressed genes
DFR: Dihydroflavonol 4-reductase
DNBs: DNA nanoballs
Dw: Dry weight
FDR: False discovery rate
F3H: Flavanone 3-hydroxylase
FLS: Flavone synthase
F3'H: Flavonoid 3'-hydroxylase
FPKM: Fragments per kilobase of exon model per million mapped fragments
FW: Fresh weight
GO: Gene ontology
HPLS: High-performance liquid chromatography
LDOX: Leucoanthocyanidin dioxygenase
LAR: Leucoanthocyanidin reductase
LIT: Linear ion trap
MWDB: MetWare database
MRM: Multiple reaction monitoring
PAL: Phenylalanine/tyrosine ammonia-lyase
PBS: Phosphoric acid buffer solution
PCA: Principal component analysis
Q3: Product ions
*p*: P-value
QC: Quality control
RAFS: Raffinose synthase
RWC: Relative water content
RT: Retention times
HCT: Shikimate O-hydroxycinnamoyltransferase
ssCir DNA: Single-strand circle DNA
TFC: Total flavonoid content
C4H: Trans-cinnamate 4-monooxygenase
TF: Transcript factor
QQQ: Triple quadrupole
QTRAP: Triple quadrupole-linear ion trap mass spectrometer
Tw: Turgid weight
VIP: Variable importance in project
