# Supplementary material for: Transcriptional modification and the accumulation of flavonoid in the leaves of Cissus rotundifolia Lam. in respond to drought stress
Source: Stress Biol. 2025 Mar 10;5(1):19. doi: 10.1007/s44154-024-00205-6 (PMC11891113; doi:10.1007/s44154-024-00205-6)
Supplement: Supplementary file 1 — Supplementary Material 1. [file 44154_2024_205_MOESM1_ESM.docx]

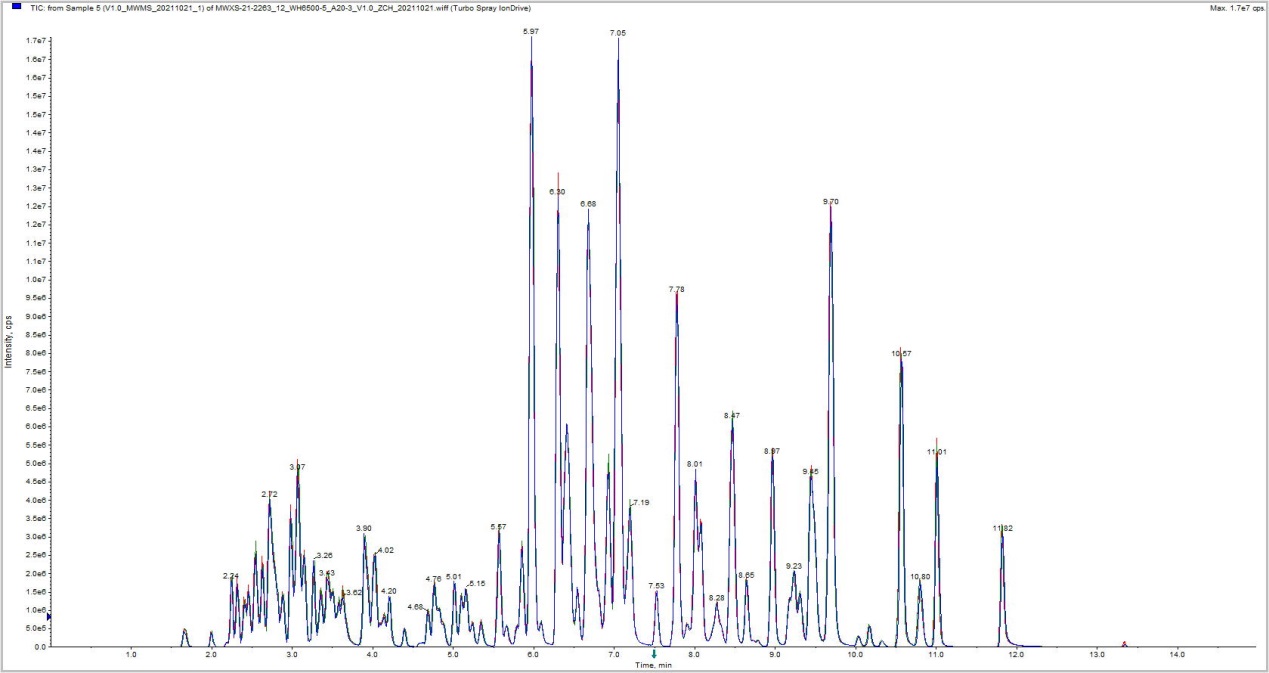


Figure S1. Total ion flow chromatogram. The x-axis represents the retention time and the y-axis the ion intensity (cps).


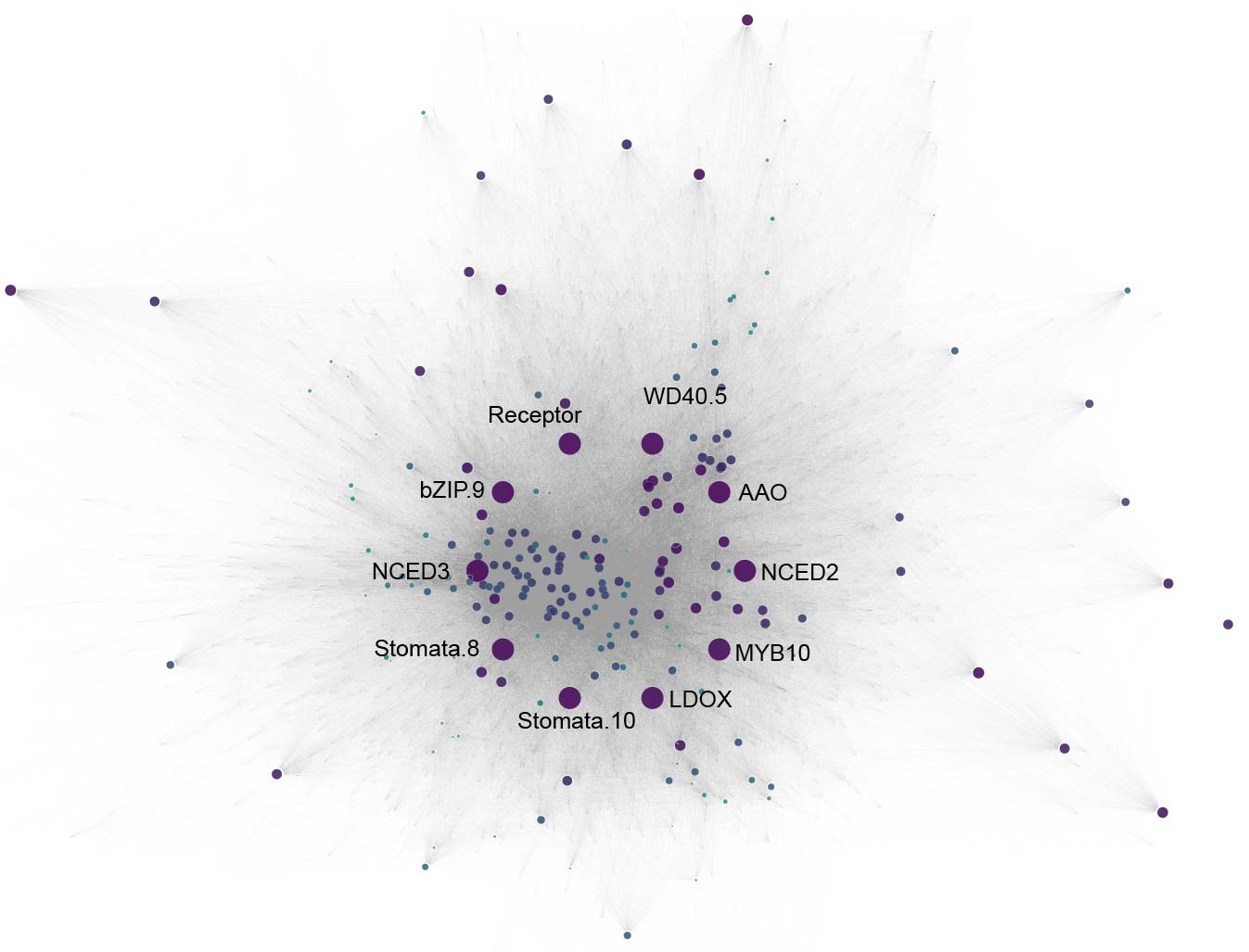


Figure S2. Top 10 hub-genes of the co-expression network constructed from all differentially expressed genes (DEGs).


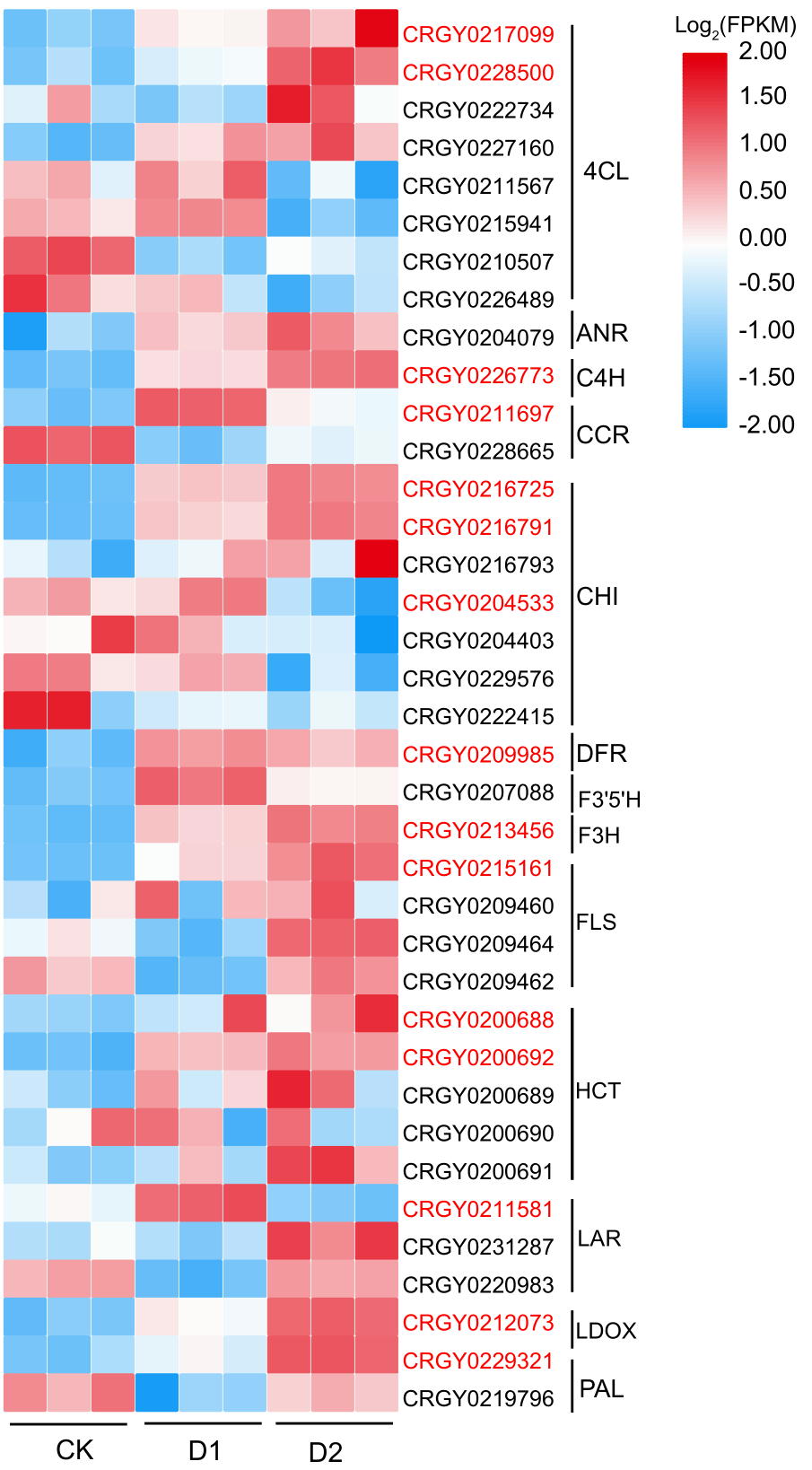


Figure S3. The expression of genes in the flavonoid biosynthesis pathway. The red letters represent differentially expressed genes.


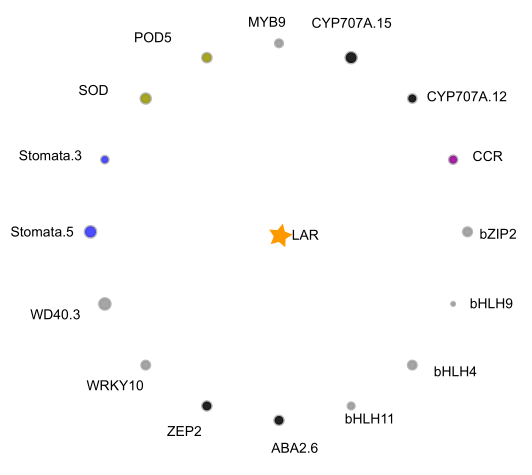


Figure S4. DEGs directly interacted with *LAR* (*CRGY0211581*). The orange, grey, black, green, blue, brown, and pink circles represent DEGs in flavonoids biosynthesis, transcription factors, ABA biosynthesis and signaling pathway, photosynthesis, stomatal movement, antioxidant enzymes and protein kinases, respectively. The size of circles represents the connectivity of the genes.
